# Crime and coronavirus: social distancing, lockdown, and the mobility elasticity of crime

**DOI:** 10.1186/s40163-020-00121-w

**Published:** 2020-07-06

**Authors:** Eric Halford, Anthony Dixon, Graham Farrell, Nicolas Malleson, Nick Tilley

**Affiliations:** 1 Lancashire Constabulary, Lancashire, UK; 2 University of Leeds, Leeds, UK; 3 University College London, London, UK

**Keywords:** COVID-19 and crime, Mobility and crime, Movement and crime, Google COVID-19 Community Mobility Reports, Mobility elasticity of crime, Mobility theory of crime

## Abstract

Governments around the world restricted movement of people, using social distancing and lockdowns, to help stem the global coronavirus (COVID-19) pandemic. We examine crime effects for one UK police force area in comparison to 5-year averages. There is variation in the onset of change by crime type, some declining from the WHO ‘global pandemic’ announcement of 11 March, others later. By 1 week after the 23 March lockdown, all recorded crime had declined 41%, with variation: shoplifting (− 62%), theft (− 52%), domestic abuse (− 45%), theft from vehicle (− 43%), assault (− 36%), burglary dwelling (− 25%) and burglary non-dwelling (− 25%). We use Google Covid-19 Community Mobility Reports to calculate the mobility elasticity of crime for four crime types, finding shoplifting and other theft inelastic but responsive to reduced retail sector mobility (MEC = 0.84, 0.71 respectively), burglary dwelling elastic to *increases* in residential area mobility (− 1), with assault inelastic but responsive to reduced workplace mobility (0.56). We theorise that crime rate changes were primarily caused by those in mobility, suggesting a mobility theory of crime change in the pandemic. We identify implications for crime theory, policy and future research.

## Introduction

In response to the coronavirus (covid-19) pandemic, governments around the world legislated for the cessation of non-essential contact. With the introduction of social distancing and lockdowns, it was soon apparent that the unanticipated effects upon crime could be dramatic (Farrell and Tilley 2020, Ashby 2020, Bump 2020, Mohler et al. 2020). Here we study the effects on crime in the days leading up to, and following, the introduction of a national stay-at-home lockdown. While we focus on one UK police service area, the methodological approach may be more broadly applicable, and the substantive findings of relevance for comparisons both to other regions of the UK and other countries with similar socio-demographic and economic profiles.

The nature of the dramatic changes to mobility that occurred allow us to approach the study as a natural experiment. We use crime data spanning 5 years to compare rates in 2020 to what would have been expected based on trends from previous years. In addition, we use Google COVID-19 Community Mobility Reports to compare area-based mobility to crime. Specifically, we compare mobility change in the retail sector to changes in shoplifting and other theft, mobility change in residential areas to burglary dwelling and theft from vehicles, and mobility in retail and recreation areas to changes in assault. This allows us to calculate the mobility elasticity of crime (MEC) as the percentage change in crime due to a one percent change in mobility.

Our approach is informed by the theoretical perspectives of crime science, particularly the lifestyle and routine activities approaches (Hindelang et al. 1978, Cohen and Felson 1979) that identify crime opportunities as central (Clarke 2012). We view mobility as a core determinant of the level of crime opportunities. Changes to mobility affect lifestyles and the likelihood of interaction between potential targets (including victims) and potential offenders, and the likelihood of surveillance and potential guardianship by others. In theory, covid-19 policies to restrict movement will affect different crime types in different ways (Farrell and Tilley 2020). For instance, increased time spent in the home might be expected to increase the opportunities for domestic violence and child abuse to occur, because they are often committed by parents or guardians, and potential victims and offenders are spending more time together. At the same time, however, increased time spent in the home might increase guardianship and surveillance against burglary. Reduced attendance at workplaces would be expected to reduce workplace harassment, and reduced travel on public transport would be expected to reduce the many types of crime that occur on such transport or around transport stations. Widespread closure of shops would be expected to reduce shoplifting. With people spending greater work and leisure time online, the opportunity for crimes to occur via the increases in virtual mobility. Hence changes to mobility would not impact uniformly but, rather, different types of crimes would be affected in different ways in different contexts, and we explore some specifics further in what follows.

The timeline and context for the study is as follows. On Wednesday 11 March 2020, the World Health Organisation (WHO) declared covid-19 a global pandemic.^1^ Five days later, on *Monday 16 March*, the UK government recommended nationwide cessation of all non-essential travel, followed by, on *Friday 20 March*, an announcement that all bars, cafes, restaurants, and gyms were required to close that day. On *Monday 23 March*, a national ‘lockdown’ was announced. Lockdown rules required everyone to stay home at all times with four exceptions; Exercise (alone or with members of the same household); Shopping for basic necessities; Any medical need, including providing care for a vulnerable person, and; Travel to or from work, but only when a person cannot work from home (Cabinet Office 2020). These four dates are shown as vertical lines in timeline charts in this study, and are labelled in Fig. 2.

We find distinct declines in many recorded crime rates in the 2 weeks before, and in the period immediately following lockdown. The sequencing of the onset of these declines tracked the timeline of events, but with different crime types responding to different types of mobility restriction at different times. While personal theft and theft from vehicles declined from 11 March, shoplifting and assaults declined from the introduction of restrictions on non-essential travel from 16 March, while public disorder and criminal damage declined from the closure of bars, restaurants and other such facilities on 20 March. We find preliminary evidence of pre-lockdown spikes in shoplifting, likely facilitated by the extra cover in crowded stores, and a lesser spike in assaults immediately before the lockdown, likely due to anticipatory ‘last chance’ socialising. The first week of lockdown brought more substantial decreases in many types of recorded crime.

We develop a metric to compare the changes in crime and mobility, which we term the mobility elasticity of crime. We find shoplifting responsive (if technically inelastic) to change in mobility in the retail sector, and burglary highly responsive to increased mobility in residential areas. We find, and vehicle-related theft responsive, assault inelastic though still somewhat responsive to changed mobility in the workplace sector, and in residential and retail/recreation areas, respectively. The primary conclusion of this study is that changes to mobility were the primary cause of changes to the rates of many types of crime in the early stages of the pandemic.

## Method

### Study area and crime data

This study uses 5 years of daily counts of recorded crime data from a UK police service covering over 5000 square kilometres (2000 square miles) with a population in 2020 of around 1.5 million. The service employed over 5000 persons with nearly 3000 police officers and over 2000 police staff.^2^ Crimes recorded between 8 March and 02 April 2020 are compared to the expected rates based on crimes recorded in the previous 4 years.^3^

The police service’s recorded crime data was assessed as ‘Good’ in a 2019 report by Her Majesty’s Inspectorate of Constabularies and Fire and Rescue Services covering data since 2017 (HMICFRS 2017, 2019). The report estimated recording accuracy of the Police Force at 93.3 percent with a confidence interval of ± 1.48 percent.^4^ The models we used, described next, account for variation in data quality by increasing the confidence intervals, making statistically significant changes harder to detect. The overall effect is therefore to make the study findings conservative, that is, under-stated.

### Crime rate model building and analysis

The expected level of crime in 2020 was forecast using models that drew on data from previous years. Five years’ of recorded crime data was used for the period covering the latter end of February, the whole of March and the beginning of April.^5^ The same 5-week period was used from each year, to minimize seasonal confounds. The 5 weeks were aligned by day of the week to minimize potential effects of differences due to day of the week. Specifically, data for each year was aligned by the third Friday in March. The data was then trimmed to provide a 35-day snapshot that broadly included the last week of February and all of March for each year, the process governed by the need to have whole weeks and to exclude school or public holidays (avoiding possible confounds because the dates often vary by year). The data were collected in mid-April to alleviate the delay in crime reporting that can occur. That is, although the crime series data end on 02 April, they were compiled in the days after that date, which should account for any lag between crime events taking place and being recorded by the police.

Time series models were built using the 2016 to 2019 data and the first week of records from 2020, the latter to account for the longer-term national crime decline. The remainder of the 2020 data, from 06 March to 02 April 2020, comprised the Test data, that is, the actual rates for comparison to the expected rates from the model.

For model building, the Training data were transformed into a time series with frequency seven. Trend, daily effects and weather effects were removed by applying separate linear regressions for each effect, and the coefficients used in the models. A piecewise linear model approach was adopted (Chatfield 2016) which allowed trends to be analyzed sequentially in order to mitigate breaks in data, changes to reporting, and other changes in trend. Hence, while seasonality and weather effects relate to the entire reporting period, trend information was used in a more targeted manner so that expected/forecast values contained the appropriate trends. Potential weather effects were represented by the maximum daily temperature and amount of rainfall (mm) (Historical Weather data 2020). Interaction affects within weather and day of the week were not statistically significant, which meant that the simpler models described below were adopted. The resulting residuals from the detrended and deseasoned models were analysed with ARIMA time series models, appropriate models then selected using an automated function in R (Hyndman et al. 2020). The automated function used a step-wise selection algorithm (Hyndman and Khandakar 2008) based upon Akaike information criterion (AIC) to iteratively determine the coefficients for the ARIMA model which best explained the deseasoned and detrended residuals. The resulting ARIMA model was used, along with the daily effect, the weather effect and trend, to produce a forecast value for each crime type with an associated 95% confidence interval.^6^ Hence the general equation for the crime rate was:

$$X_{t} = \mu \left( t \right) + s\left( t \right) + w\left( t \right) + \epsilon_{t}$$

where $X_{t}$ is the Crime rate, $\mu \left( t \right)$ is the trend, $s\left( t \right)$ is the daily effect, $w\left( t \right)$ is the weather effect and $\epsilon_{t}$ are the random errors. All at time t.

### Mobility data

Previous studies have used various data sources to explore the role of ambient populations upon crime rates (Andresen 2006, Malleson and Andresen 2015a, b, Boivin 2018, Kounadi et al. 2018, Hipp et al. 2019, Johnson et al. in press). Due to the recency of the period under study, these data were unavailable. To examine mobility, we use data extracted from the Google COVID-19 Community Mobility Reports^7^ .

The mobility reports “show how visits and length of stay at different places change” based on data from phone users who have turned on their location history (Google 2020). The reports are aggregations of locations over time and compare recent activity to a historical baseline. The six locations or spheres of activity used are: residential, retail & recreation, workplaces, grocery stores,^8^ parks, and transit stations. A limitation of the data is that the baseline is the median value, for the corresponding day of the week, during the 5-week period Jan 3–Feb 6, 2020 (Google 2020). This means that the mobility data baseline comparison is less rigorous than that for our crime data (and something that future research should seek to overcome).

We compared changes in mobility and crime to calculate the mobility elasticity of crime (MEC) as a metric of the change in crime in response to a change in mobility. The MEC draws upon the concept of the price elasticity of demand, which gauges the effect upon consumer demand in response to the change in the price of a good.

To operationalise the MEC, we identified crime-mobility combinations where there were evidential and theoretical grounds to expect a relationship. Around half of the recorded theft in the study area was shoplifting, which is located largely at retail areas. Around two-thirds of personal theft occurred in or around a shop or supermarket, a public entertainment area or the street, or inside a pub according to the most recently available Crime Survey for England and Wales data (CSEW, ONS 2019).^9^ Consequently, we compared shoplifting and other theft to mobility in retail and recreation areas.

Burglary dwelling occurs in residential areas, so they were our most obvious pairing. Around a third of assaults occurred at or around the workplace, but of those where the location could be grouped into one of the mobility area categories, the workplace accounted for over half (CSEW 2019a). So we paired assaults with workplace mobility while recognising the potential limitations^10^ . Over three quarters of theft from vehicles occurred at or around the home according to the most recent CSEW findings, so we compared trends in theft from vehicles to those in residential area mobility.

## Results

### Crime rates and mobility rates

**Fig. 1:**
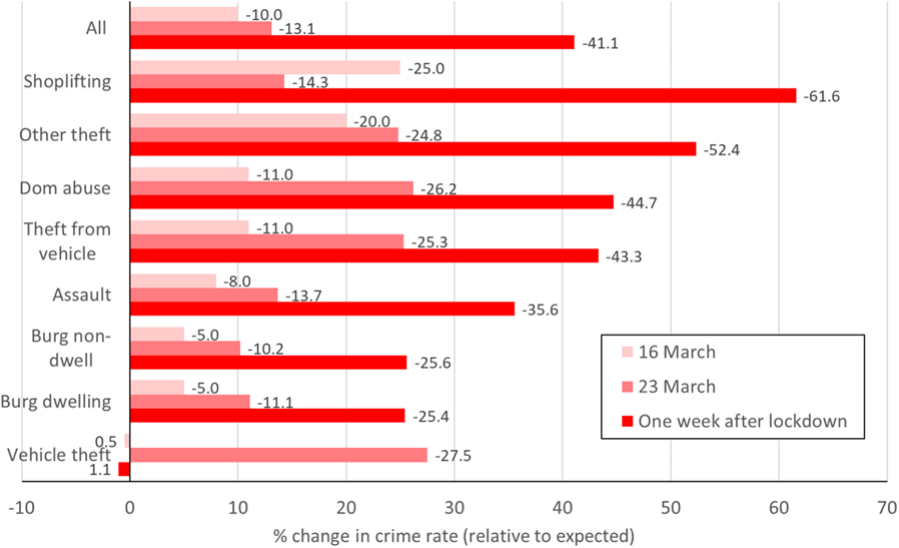
Percent change in crime by week after lockdown

**Fig. 2:**
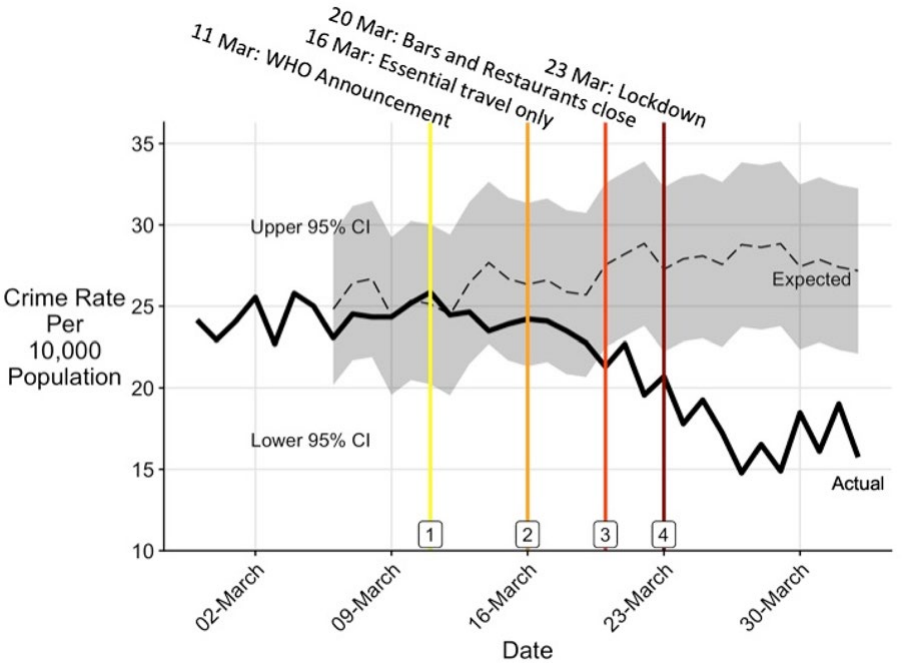
All crime—comparison of March 2020 actual and expected rates

**Fig. 3:**
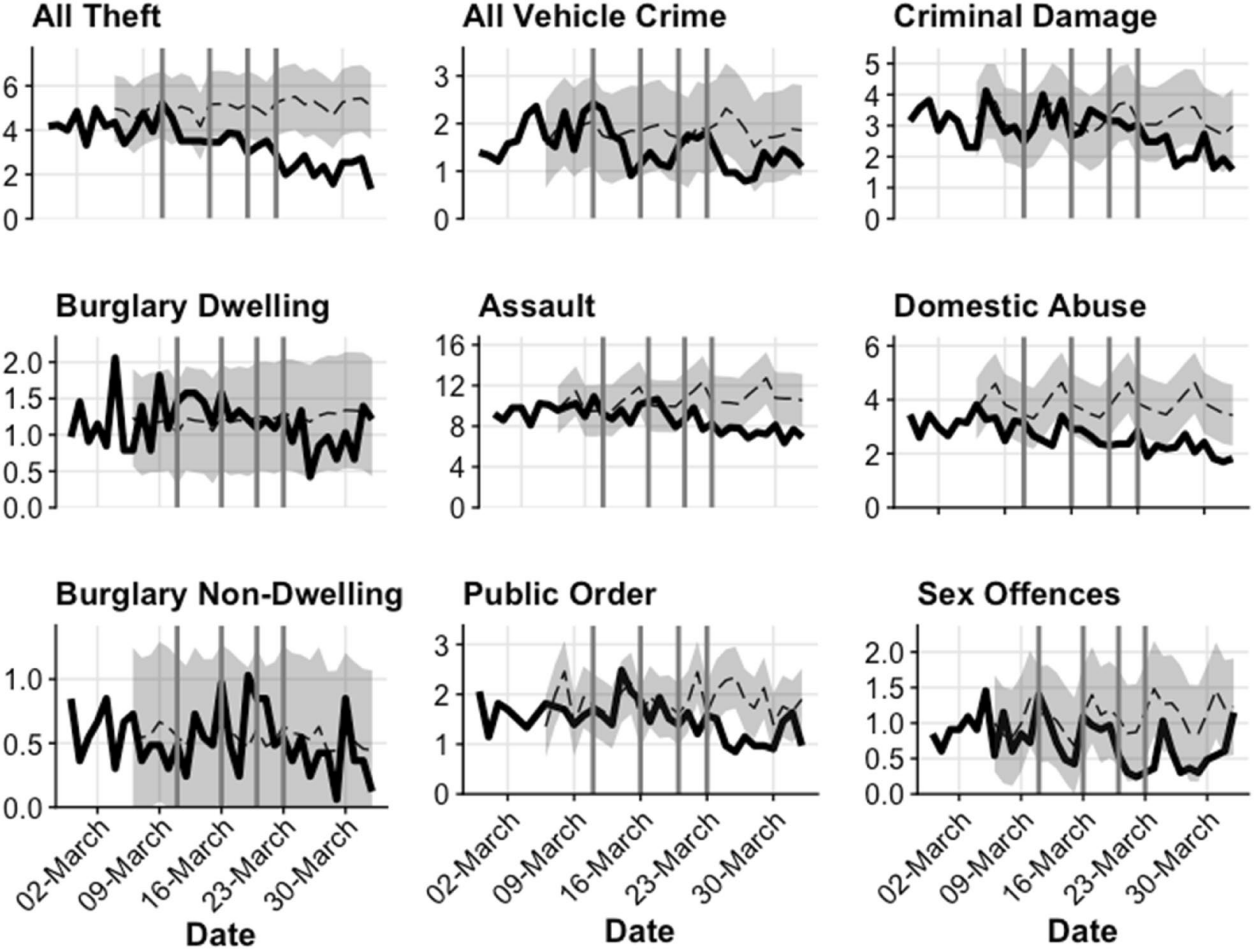
Recorded property crime rates per 10,000 population—comparison of March 2020 to expected rate

**Fig. 4:**
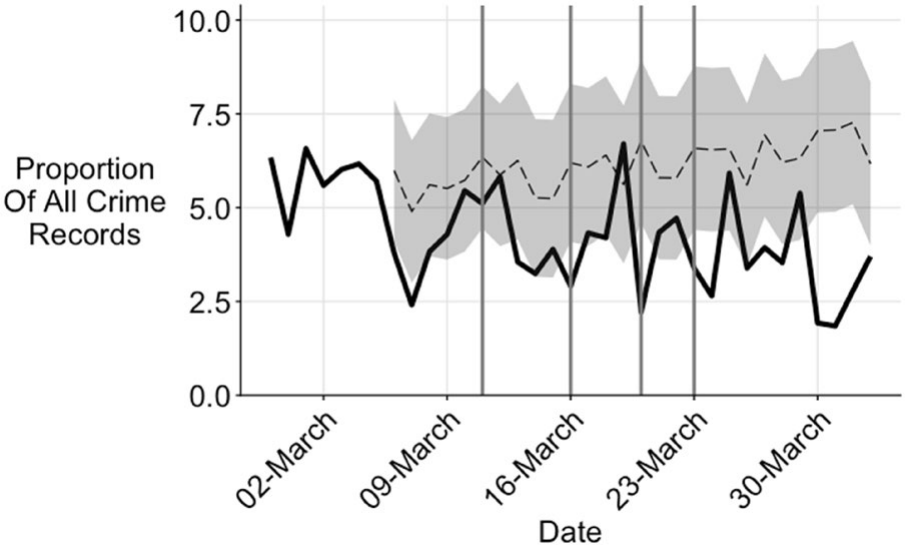
Vulnerable child indications as proportion of all crime

A summary of the changes to crime rates for the ‘pre-lockdown’ period 11-23 March, and by one week after lockdown, are shown as Fig. 1. Note that readers should interpret Fig. 1 in the context of the confidence intervals for individual crime types in Figs. 2, 3, 4 and Table 1. By 1 week after lockdown, all crime types had declined except for theft of motor vehicles. Theft and shoplifting had declined by more than half, assault, theft from vehicles and domestic abuse between a third and a half, and burglary by a quarter.

**Table 1:** Change in crime rates by 02 April

Crime type	Days since 16th March	
	Below expected rate (%)	Outside confidence interval (%)
All recorded crime	100	78
All Theft	100	72
Domestic Abuse	100	61
Vulnerable child	94	56
Assault	89	50
Public Order	94	44
Shoplifting	89	39
Criminal Damage	89	28
Theft from vehicle	100	17
Sex crimes	100	11
All burglary	56	6
Burglary non-dwelling	72	0
Burglary dwelling	67	0
Theft of vehicle	44	0

‘Vulnerable adult’ category excluded due to data quality

The ‘all crime’ category declined after 11 March (Fig. 2), and was statistically significantly different from the expected rate by 21 March, remaining so for the duration of the study period (Table 1). The decline had levelled-off by late March. All individual types of recorded crime began to decline before lockdown, with variation in the timing and extent, as discussed below. The crime-specific results that follow are chronological by date of onset of identifiable change, in order to correspond with Table 3 which is discussed later.

*Theft* declined from the WHO ‘global pandemic’ announcement of 11 March and fell 20 percent by 13 March. A decline in *shoplifting* began around 16 March when non-essential travel ceased, but was particularly pronounced after lockdown, becoming statistically significantly low and remaining so through early April (Fig. 3 and Table 1). By the end of March, 1 week after lockdown, shoplifting had declined 62% and all theft 40 percent. In previous years there had been a sharp decline in shoplifting on Sundays, which was less marked in March 2020. *Theft from vehicles* also declined from 11 March, and halved by mid-March (while *theft of vehicles* declined later, following lockdown). Recorded *sex offences* declined from around 13 March, the trend continuing through March.

The ‘*vulnerable child*’ category refers to any type of recorded crime where a child was flagged as vulnerable. This means that the 41 percent decline in all recorded crime would, other things equal, produce a similar decline in vulnerable child records. Hence a preferable measure is change is the *proportion* of recorded crimes with a vulnerable child indicator, shown as Fig. 4.

*Assaults* declined from 16 March, continuing through March, but the weekend increases of previous years were absent. Recorded *domestic abuse* declined from around 16 March onwards, and had declined (statistically significantly) by over 40 percent 1 week after lockdown, with weekend peaks also less prominent.

*Criminal damage* declined after 20 March. The apparent resurgence in criminal damage around 30 March–may reflect delayed reporting after a weekend. *Public disorder* also declined from 20 March, with previous weekend increases absent, with a possible early April increase.

*Burglary* declined following lockdown on 23 March, falling by half over the next week. A possible resurgence in burglary dwelling by early April could reflect a reporting increase after the weekend. *Theft of vehicles*, while numerically small as a daily count, declined from the lockdown of 23 March.

**Fig. 5:**
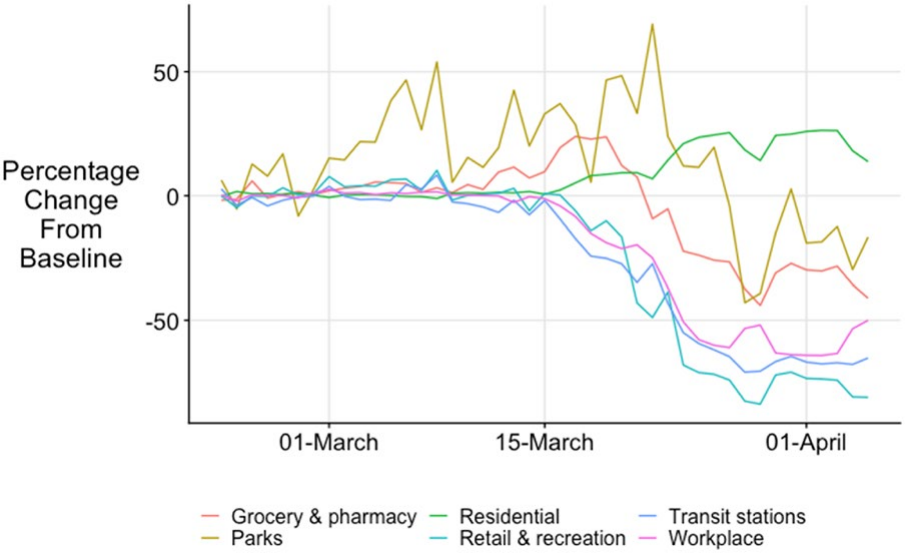
Mobility changes by area relative to baseline expected rate

Changes in mobility in the six types of area, relative to the baseline, are shown in Fig. 5. That around residential areas increased from 16 March, and 1 week after lockdown there was around 25 percent more mobility than expected in residential areas (Fig. 5). Movement around workplaces declined rapidly from 16 March and had fallen more than 50 percent by 1 week after the 23 March lockdown. Mobility around retail and recreation areas decreased after 16 March and had declined by three-quarters compared to the expected rate by 1 week after lockdown. There was *increased* movement around grocery stores in mid-March, followed by a substantial decline beginning before lockdown on 23 March, with a decline of around a third by 1 week after lockdown. There was greater than usual movement around parks for much of March, but a sharp decline by late March. Mobility around transit stations declined from 16 March and had declined by well over half by 1 week after lockdown.

### Mobility elasticity of crime

**Fig. 6:**
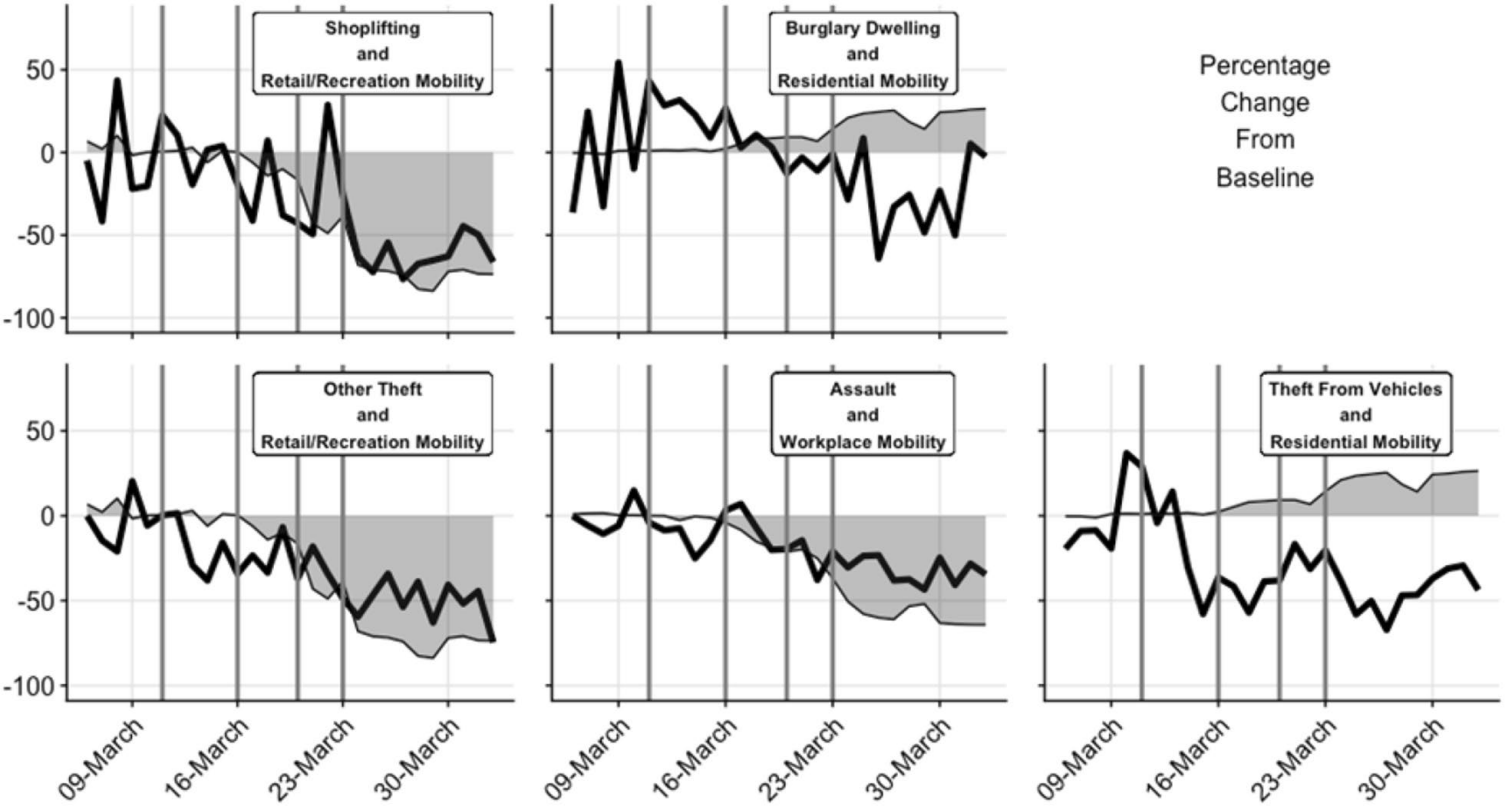
Comparison of changes in crime and mobility (relative to baseline expected rates) for crime-mobility pairs used in elasticities analysis and shown in Table 2

The visual comparisons of the deviation from baseline expectations for the crime type and mobility comparisons are shown as Fig. 6. The resulting mobility elasticities of crime are shown as Table 2.

**Table 2:** Mobility elasticity of crime by crime type

Crime type	% change in crime	Location	% change mobility	Mobility elasticity of crime (MEC)
Shoplifting	− 61.58	Retail & recreation	− 73.65	0.84
Other Theft	− 52.36	Retail & recreation	− 73.65	0.71
Burglary dwelling	− 25.44	Residential	24.84	− 1.04
Assault	− 35.56	Workplace	− 63.18	0.56
Theft from vehicle	− 43.32	Residential	24.84	− 1.74

Visual inspection of Fig. 6 suggests that the timing and magnitude of change to shoplifting and other theft tend to coincide with those to mobility in retail and recreation areas. The respective MECs of 0.84 and 0.71 are inelastic but suggest that both are quite responsive. That is, each one percent reduction in mobility produced slightly less than a one percent change in theft.

The timing and magnitude of change to burglary dwelling and residential area mobility fit quite well (Fig. 6), but inversely. That is, increased residential area mobility corresponded with decreases in burglary dwelling. The MEC of − 1.04 is effectively unitary which suggests that a one percent increase in residential area mobility produces a one percent reduction in residential burglary.

The timing and magnitude of change to assaults and workplace mobility do not coincide quite as well as those discussed so far. After lockdown, workplace mobility declines proportionally more than assaults (Fig. 5). The MEC of 0.56 supports this interpretation, suggesting that each one percent decline in workplace mobility produces a 0.56 percent decline in assaults.

The weakest relationship examined here is that between the timing and magnitude of change to theft from vehicles and residential area mobility. Theft from vehicles declines earlier, more rapidly and to a greater extent than residential area mobility increases (Fig. 5). The MEC of -1.74 is elastic, suggesting that theft from vehicles declines more than proportionally in response to increases in residential area mobility, but the lack of good visual fit between the two suggests this should be interpreted with caution.

## Discussion

Area-based mobility and crime types are discussed separately first. The two aspects are then brought together in a discussion of their relationship.

Changes in area-based mobility levels in the study area fit largely with expectations and the timeline of covid-19 and related policies (Beadsworth 2020). Mobility in most areas declined in the lead-up to or shortly following lockdown, reflecting restrictions on movement. The increased movement in parks prior to lockdown probably reflects the warmest March since 1957 (BBC 2020a). The short-term pre-lockdown increase in mobility at grocery stores reflects preparatory purchases and stockpiling. Mobility around areas of retail and recreation, workplaces and transit stations began to decline from 16 March, reflecting the national advisory to cease non-essential travel and work at home.^11^ Mobility trends in these three areas are similar, reflecting the interconnectedness of the activities that they represent. The increase in mobility around residential area occurs as people remained at home but undertook permitted local exercise.

Variations in the onset of change by crime type correspond largely with the timeline of change relating to covid-19 from 11 March onwards: a summary of the timeline and onsets of change is given as Table 3. The timing of the onset of decline in the ‘all crime’ category coincided with the WHO announcement of a ‘global pandemic’ on 11 March. Theft, which as a higher volume crime contributes more to the ‘all crime’ category for present purposes, declined from 11 March (Fig. 3). From 16 March, as social distancing was more formally introduced, and non-essential travel reduced, the decline in mobility at retail and recreation areas, workplace areas and transit stations began (if slowly), and so too did the declines in assaults. The closure of bars, restaurants, and gyms from 20 March largely coincides with the onset of declines in criminal damage and public disorder.

**Table 3:** Timeline dates with changes in mobility and crime

Period	Date & event	Mobility change	Crime change
1	11 March 2020: WHO ‘global pandemic’ announcement	*Increased* mobility around grocery stores until lockdown	‘All crime’ decline begins; Declines begin for theft (other theft), theft from vehicles and recorded sex offences
2	16 March 2020: No none-essential travel—‘formal social distancing’	Onset of *decreases* at Retail & recreation, and Workplaces. Onset of *increase* at residential areas	Assaults decline begins (subsequent weekend spikes were absent). Shoplifting decline begins
3	20 March 2020: Closure of bars, restaurants. gyms etc		Criminal damage decline begins (weekend spikes absent); Public disorder decline begins
4	23 March 2020: National lockdown	*Decreased* mobility around grocery stores begins	Burglary decline begins; Theft of vehicles decline begins

Shoplifting had declined dramatically by the first week of lockdown. Most retail (non-grocery) shops and stores were closed at lockdown, dramatically reducing shoplifting opportunities. Supermarkets and other stores that remained open promoted social distancing, which may have discouraged shoplifting. It is possible that stores that remained open experienced an *increase* in shoplifting. However, if they did, then it was at most a small fraction of total potential crime displacement, consistent with other findings (Johnson et al. 2014).

Non-shoplifting other thefts declined earlier than shoplifting. By one week after lockdown, other thefts had declined around half, that is, extensively but not as much as shoplifting. The overall decline fits with expectation that fewer potential victims of theft were available in retail and recreation areas, as well as on public transport and at workplaces where mobility had declined. The proportionally greater reduction in shoplifting than other theft is explained by the closure of almost all stores where shoplifting could take place, whereas a greater proportion of opportunities for other thefts remained elsewhere.

Recorded burglary dwelling declined a quarter by the first week of lockdown. By any normal measure this was a large decline. It is likely explained by increased home guardianship and surveillance (including by neighbours) as residents stayed home. However, the decline is less than that in shoplifting and arguably not as extensive as might have been expected. The mobility elasticity of burglary, being unitary, sheds light on this issue. While burglary was highly responsive to change in residential area movement, that movement only increased 25 percent. If guardianship and surveillance increased proportionally with movement then perhaps the 25 percent decline in burglary is readily explained.

The one quarter reduction in burglary of non-dwellings also fits with the explanation for burglary dwelling. While many commercial premises would be closed and locked, they would not benefit from the guardianship of increased occupancy (except natural surveillance from those overlooking such premises). Decreased movement of offenders might have played a greater role, but further research is needed to investigate that possibility further.

Elsewhere we suggested that domestic violence would be expected to *increase* under lockdown (Farrell and Tilley 2020). Other things equal, increased interactions between potential victims and offenders in domestic setting would represent an increase in the number of opportunities for domestic abuse. It is possible that the drop in recorded domestic abuse found here is genuine, and could have come about as a response to a decrease in external pressure placed on a household, for example, through reduced alcohol consumption in bars or shorter working hours. However, there is increasing evidence that an increase in domestic violence has occurred (BBC 2020b, The Guardian. 2020). The 40 percent decline in recorded domestic abuse observed here is, we suggest, more likely to reflect a reduction in reporting and recording. There are multiple routes by which this could occur. In particular, social distancing may well have increased the difficulty of reporting domestic abuse if the offender remained on scene and could not be separated. If this is the case then there is evidence of a worrying increase in the number of victims not having access to the help and assistance they need, and that this is not conveyed by the recorded crime statistics.

We interpret the pronounced decline in theft from vehicles as reflecting reduced use of vehicles. This means that fewer vehicles were parked in city centres during the working day, or in other non-home locations such as entertainment districts at other times, where they may have been vulnerable to theft. Previous research has shown that vehicles parked at home, particularly those on driveways or in garages, are at reduced risk of theft (Clarke and Mayhew 1994).

The small numbers and greater daily variability in theft of vehicles makes it harder to interpret the trend. However, it is possible that, apart from the days immediately following lockdown, there is a less discernible effect upon theft of vehicles. The major reductions in vehicle theft in recent decades means that proportionally more of it has become professional or organised in recent years, requiring offenders with greater skills. This includes the commission of car-key burglaries, RFID-interception, and immobilizer-bypass technologies (Wellsmith and Burrell 2005, Brown 2016). If these resourceful offenders were more likely to ignore the lockdown, and perhaps to view it as an opportunity to search for desirable on-street vehicles with less natural surveillance due to reduced footfall, then it is possible that theft of vehicle would not decline to the same extent as the less-skilled crime of theft from vehicles. These are issues that further research and larger datasets, discriminating between age, make and recovery of stolen vehicles, might explore.

The workplace mobility elasticity of assault of 0.56 indicates that each one percent decline in mobility in and around the workplace produces around half the impact in terms of reduced assaults. Recall, however, that only around a third of assaults occurred at or around the workplace, so it would not necessarily be expected to be a strong relationship. More generally, the declines in assault, criminal damage and public order offences found here are largely consistent with the declines in outdoor activities at retail and recreation areas. This includes attendance at sporting events and other forms of recreation and entertainment including the closure of pubs and bars.

The decline in the proportion of crimes with a vulnerable child indicator may have occurred as a result of a change in the crime mix, as a result of reduced likelihood of reporting and recording of vulnerable children, as a result of an actual decline, or some combination of these possibilities. Sex crimes are rarely reported and recorded, making interpretation of the trend difficult. If recorded sex offences are more likely to be outside the home, then reduced mobility in relevant areas may well explain this reduction, but further research should examine such issues.

### Study Limitations

Some types of crime are less likely to be reported to the police than others (Office for National Statistics 2020). For this study, reporting and recording will also have been affected by circumstances particular to the pandemic. This could include the effect of police staff absences due to illness or self-isolations, the need to reduce risk of infection in police work, the closure of some custody suites, and increased single-crewing of police vehicles to promote social distancing. Victim surveys may, in time, provide insight into the extent to which this occurred.

A potential limitation of the crime rate modelling was that, if the effects of covid-19 spread earlier than we assumed then the effect would be included in the week of 2020 data used in the forecasting model. It is also possible that other variables influenced the 2020 crime rates differentially in comparison to previous years, the most likely effect of which would be to inflate the confidence intervals for the present analysis. Both these possibilities would make our estimates more conservative, under-stating the effect of change attributable to covid-19. In general, however, the size of the crime rate change in 2020 and its correspondence with both the timeline, mobility change and the fit with theoretical expectation, indicates that change relating to the covid-19 pandemic played the primary role.

The Google Covid-19 Community Mobility Reports data was grouped into six areas, which were imperfect for present purposes. For example, it is likely that a comparison of shoplifting to mobility in some combination of the grocery and retail areas would be preferable. Proportional weighting of mobility areas according to areas where crimes occur might improve the quality of the match. Ideally, both datasets would be georeferenced to allow more precise spatial analysis. Hence further research would benefit from more precise geographical linking of the mobility and crime data. In addition, the baseline comparison group for the mobility data was earlier weeks in 2020, which is less robust than a baseline comparison group from several previous years of data: it is to be hoped that further research can continue to develop these comparisons.

The aggregate mobility data used here did not distinguish between the movement of potential offenders and potential victims. Perhaps future research, using retrospective tracking of mobility at the individual level, can develop such comparisons. For example, the movements of known offenders and those arrested during the course of events examined here, could be separately traced to determine the extent to which they varied from aggregate movement patterns and those of other sections of the population. To that extent, mobility tracking has potentially widespread implications for the study of crime.

This study did not address all types of crime. This includes drug-related offences and organised crimes of various types. We recognise that new opportunities have arisen for fraud and theft of medical equipment, while many online crime types are likely to have been facilitated by increased online remote working and leisure activities, but were outside the scope of this study. In the context of mobility though, physical movement restrictions can, via increased online work and leisure, increase the virtual movement necessary for internet-related crimes from online child sexual abuse to fraud, and this suggests how the present study may shed light on changes to other types of crime.

## Conclusion

Most governments around the world restricted the movement of people through some combination of social distancing and lockdown, as part of efforts to tackle the coronavirus pandemic. This produced a range of unintended consequences, including upon crime.

This study examined the effects on crime in the days leading up to, and following, a lockdown. Changes were found to largely fit with predictions, based on crime science theories, set out before much of the evidence was available (Farrell and Tilley 2020)^12^ . Our principal conclusion here is that variations in the timing and trajectory of crime rate changes largely reflect those in mobility that occurred in response to covid-19 and policies to address it. That is, we suggest a mobility theory of crime change in the pandemic.

Many crimes continued to be committed during these early stages of the pandemic. For example, shoplifting declined 60 percent which means it still occurred at 40 percent of its expected level. Here the mobility elasticity of crime offers useful explanatory insight. For example, the inverse unitary relationship between residential area mobility and burglary dwelling fits with how mobility changes in residential areas were relatively smaller than those in retail and recreation areas, and so too were the effects on crime.

In addition to the need for replication and extension of the work begun here, there are other issues for further research. Clearly, further study of the mobility-crime relationship is needed, amongst which the potential to examine offender and victim mobility may prove particularly fruitful. More generally speaking, alternative applications of mobility reports, particularly if they can be further disaggregated, holds significant potential. Victim surveys should shed light on the reporting and recording of crime in the pandemic. We also recognise that there are various types of crime not addressed here that are also important and will have been facilitated by conditions relating to the pandemic. Changes to virtual mobility, that is, online traffic in different domains, may shed light on online crimes just as physical mobility has shed light on physical crimes here.

As we exit lockdown and mobility levels change, our theory suggests that crime will respond. Many crime types will increase as mobility increases, but a v-shape bounce-back would be negative, and policy and practice to promote an L-shaped trajectory should be encouraged. A w-shape crime trajectory might result from further covid flares-ups and lockdowns, both nationally and locally. Strategies that anticipate such changes need to be developed.

Many crime types have been in long-term decline in recent decades, particularly in developed countries. Reduced crime opportunities, mediated by improved security and reduced target suitability, has emerged as a strong explanation (Farrell et al. 2014). Crime changes relating to the coronavirus pandemic are consistent with effects upon crime opportunities of changes to mobility. That is, the relatively short-term rapid changes in crime experienced during the covid-19 pandemic appear consistent with the explanation offered for the longer-term international crime drop, but so too with increases in cybercrime, fraud and other new and emerging crimes that emerged as the result of increased crime opportunities. Policy and practice based on this insight are likely to differ from those seeking to address crime from other perspectives.

The reduced crime opportunities to which the international crime drop is attributed have been found to disproportionately reduce juvenile offending. One study suggested this has had a longer-term ‘legacy effect’ because each annual cohort of juvenile offenders was smaller, meaning fewer continued in offending over the life-course, thereby further reducing crime in the decades that followed (Farrell et al. 2015). The pandemic could, conceivably, produce a similar but smaller legacy if the 2020 cohort of potential young offender remained uninitiated, or found offending more difficult and less rewarding, because of mobility restrictions. If there is something to this, the smaller 2020 cohort of continuing offenders will produce an inadvertent beneficial legacy of covid-19 policies in years to come.

## Data Availability

Not applicable.

## Footnotes

1 We refer to covid-19 as the term used by the World Health Organisation for what is formally known as ‘severe acute respiratory syndrome coronavirus 2’ or SARS-CoV-2 according to the Committee on Taxonomy of Viruses (New Scientist 2020).

2 Lancashire Constabulary. 2020. ‘What We Are and What We Do’ Lancashire Constabulary website, at https://www.lancashire.police.uk/about-us/accessing-information/publication-scheme/who-we-are-and-what-we-do/. Accessed 06 April 2020.

3 The crime categories included in this analysis are: burglary of a dwelling (burglary dwelling) and burglary of non-dwellings (burglary non-dwelling), both categories including relevant distraction and attempted offences; theft offences which we split into shoplifting and ‘other theft’ offences (including theft of pedal cycle, theft by an employee, theft of mail, dishonest use of electricity, theft from a machine or meter, blackmail and all other theft offences); theft of motor vehicles; theft from motor vehicles; criminal damage; public disorder; violence against the person (including, assaults with injury, assaults without injury, cruelty to children, child abduction, murder, manslaughter, all assaults on a constable and all racially or religiously aggravated assault); domestic abuse; vulnerable child offences (any crime record identifying a vulnerable child). We also include a category of all recorded crime. For further specifics, readers are referred to the national *Counting Rules for Recorded Crime* (Home Office 2020).

4 There had been questions raised about the accuracy of the recorded crime data before 2018, and adjustments were made to data for some crime types to account for under-recording during this period through piecewise linear models.

5 Crime data was available for some but not the whole five-year period for criminal damage, vulnerable child indications, sex crimes and public order offences, as detailed further in the text.

6 As an additional quality control check, we ran a placebo model for 2019, and the forecasts it produced remained within confidence intervals for the duration of the study period.

7 https://www.google.com/covid19/mobility/.

8 Technically this is the ‘grocery and pharmacy’ area. In the US, for which the area categories were defined, pharmacies are often larger and more similar to UK supermarkets so, for clarity in the present context, we drop the term ‘pharmacy’ here.

9 The remainder of personal theft occurred at public transport or ‘other’ locations.

10 This excludes the CSEW locations ‘On the street’ and ‘Other’.

11 In addition, there was increased mobility around grocery stores *before* lockdown (not shown in our Figures, likely reflecting planning purchases or ‘stockpiling’ of food. There was reduced mobility around transit stations from 16 March (with timing and magnitude similar to workplace areas). There was a relatively late decline in mobility around parks, likely reflecting continued use of parks until new police enforcement powers to enforce the lockdown came into effect on 26 March.

12 Farrell and Tilley (2020) was drafted 24 March, pitched for publication 25 March, revised and published 02 April.
